# Disarrangement and reorganization of the hippocampal functional connectivity during the spatial path adjustment of pigeons

**DOI:** 10.1186/s40850-022-00143-8

**Published:** 2022-10-04

**Authors:** Mengmeng Li, Shuguan Cheng, Jiantao Fan, Zhigang Shang, Hong Wan, Lifang Yang, Long Yang

**Affiliations:** 1 School of Electrical and Information Engineering, Henan Key Laboratory of Brain Science and Brain-Computer Interface Technology, Zhengzhou University, Zhengzhou, China; 2 Department of Biopsychology, Institute of Cognitive Neuroscience, Faculty of Psychology, Ruhr University Bochum, Bochum, Germany

**Keywords:** Spatial learning, Path adjustment, Functional connectivity, Hippocampus, Pigeon, Goal-directed behaviour

## Abstract

**Background:**

The hippocampus plays an important role to support path planning and adjustment in goal-directed spatial navigation. While we still only have limited knowledge about how do the hippocampal neural activities, especially the functional connectivity patterns, change during the spatial path adjustment. In this study, we measured the behavioural indicators and local field potentials of the pigeon (*Columba livia*, male and female) during a goal-directed navigational task with the detour paradigm, exploring the changing patterns of the hippocampal functional network connectivity of the bird during the spatial path learning and adjustment.

**Results:**

Our study demonstrates that the pigeons progressively learned to solve the path adjustment task after the preferred path is blocked suddenly. Behavioural results show that both the total duration and the path lengths pigeons completed the task during the phase of adjustment are significantly longer than those during the acquisition and recovery phases. Furthermore, neural results show that hippocampal functional connectivity selectively changed during path adjustment. Specifically, we identified depressed connectivity in lower bands (delta and theta) and elevated connectivity in higher bands (slow-gamma and fast-gamma).

**Conclusions:**

These results feature both the behavioural response and neural representation of the avian spatial cognitive learning process, suggesting that the functional disarrangement and reorganization of the connectivity in the avian hippocampus during different phases may contribute to our further understanding of the potential mechanism of path learning and adjustment.

## Background

Goal-directed navigation is a kind of typical spatial navigation closely related to the migration, foraging, and homing of animals, in which the agents will make complex decisions by perceiving the important environmental information. It is very important for the survival of individuals [1]. The execution of navigation involves the planning and adjustment of the path, which is one of the principal tasks in goal-directed navigation. The mechanism of environmental perception and behaviour decision-making functions of the brain have been widely studied [2–6]. Researches show that abundant spatial information will be ultimately projected to specific brain regions for processing and integration. According to the current leading theory, the hippocampus (Hp) is mainly related to spatial navigation and the formation and storage of long-term memory [7]. The neurons in Hp are highly adaptive to process and encode the information of the surrounding world, indicating its crucial role for flexible navigation [8].

The Hp has become the most concerned target brain region in the study of spatial navigation mechanism since O’Keefe found the place cell which is considered to be the basic unit of the spatial cognitive map in Hp of rats. It seems that the activities of place cells during theta oscillation are suitable for encoding the information of current position and local trajectory on a fast time scale, which is very important for the decision-making behaviour in the space environment [9]. Before the animal starts from any starting position to the known destination in the goal-directed task, the place cell network in Hp will generate a short sequence of neural activities, encoding the spatial trajectory to be executed [10]. Further research has shown that theta oscillation (6–10 Hz) sequences in Hp are temporally compressed representations of the animal’s trajectory, reflecting the routing information next [11]. These researches in recent years have shown that Hp supports not only the representation of the current location but also the representation of the future path planning [12], contributing to a series of functions including planning, learning, the formation and maintenance of cognitive map [13]. Besides, the synchronous activities of the place cell ensembles in Hp under a series of different rhythms, such as slow-gamma rhythm and sharp-wave ripples (SWRs), also play important roles in the dynamic encoding of routing information [14, 15]. After being familiar with a specific path, the Hp enables place-related ensemble patterns to replay during subsequent states, which is believed to help animals learn the path to the goal [16]. Previous studies have shown that the Hp of birds is similar to mammals in terms of receptor architecture and functional subareas partition [17, 18]. And a series of researches have shown that the Hp of the bird plays an important role in the goal-directed behaviour, and it participates in the encoding of routing information to support the path planning and adjustment in the navigation process [19–23]. As a species of bird with excellent spatial navigation ability, the pigeon (*Columba livia*) has always been a typical model animal to study the mechanism of perception and spatial representation in navigation-related researches [24, 25].

As the oldest and the most common paradigms in animal cognition research [26], the detour paradigm refers that the current familiar path is blocked and a new non-preferred path must be found [27, 28]. The related studies by Alvernhe et al. [29, 30] shown that spatial maps in Hp alter in response to the changes of the environment when the path to the goal need to be replanned. In their experiments, rats shifted to the appropriate path very rapidly, if not immediately, in detour sessions after the preferred path was blocked. The Hp lesion experiments of the rats in the spatial navigation task also showed that the rats with Hp lesions made more errors and took longer to find the goal in the task, especially when the environment changed [31]. While all of these studies have explored how neural systems support complex spatial navigation, we still only have limited knowledge about the brain that supports path adjusting when a forced detour is required [32], especially when it comes to the related researches in birds [33]. Even more, unfortunately, most of the studies have concentrated on behavioural researches and very few studies involve the changes of neural activities inside the brain.

There may be specific changes in brain connectivity during path adjustment in the detour paradigm. Then how do the neural activities of the birds, especially the hippocampal functional connectivity, change when the current preferred path is blocked and needs to be adjusted? In the current study, we designed a goal-directed spatial cognitive task with a detour paradigm for pigeons and recorded the behavioural and neural data during the task, hoping to explorer the role of hippocampal neural activities in the representations of spatial information during path adjustment. In the task, the pigeons were trained to learn a preferred path to the goal firstly, and then the learned path would be blocked to simulate the situation that the environment changes suddenly. We predicted that the hippocampal functional connectivity of the pigeons during path adjustment would change along with the behavioural modifications. To test for the predictions, we analyzed the data from different sessions combining the behavioural and neural analysis method. The behavioural indicators and topological properties of the functional network were analyzed statistically.

## Results

### Behavioural performance during the different phases

**Fig. 1:**
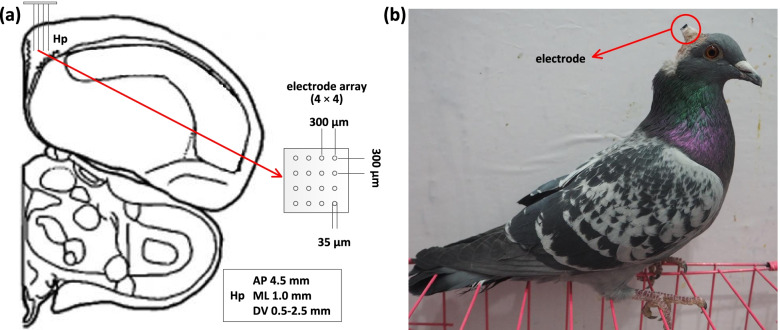
Implanting location, microelectrode array, and a pigeon with an implanted electrode. **a** Diagram of the implanting location and microelectrode array. **b** A pigeon with an implanted electrode

**Fig. 2:**
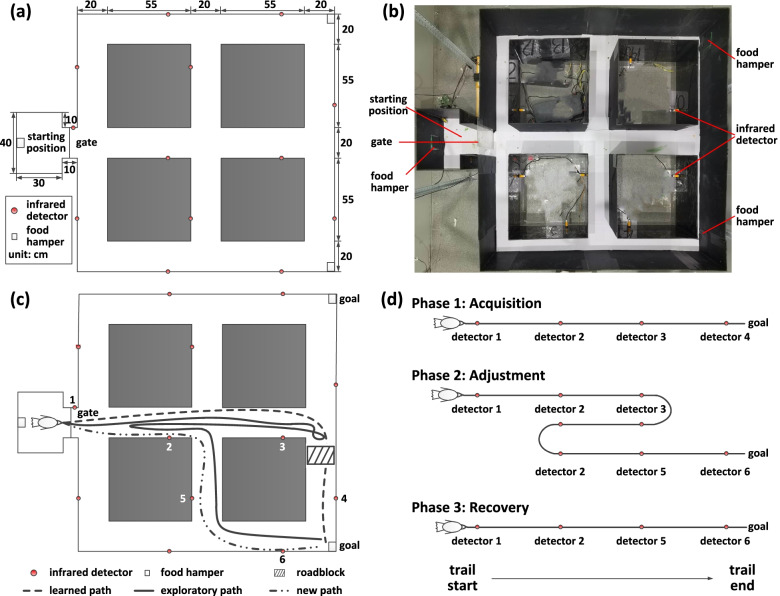
The apparatus and procedures. **a** Diagram of the maze apparatus for pigeons. **b** Photograph of the customized maze apparatus. **c** Diagram of the goal-directed spatial cognitive task. **d** Experiment procedures. The pigeons were trained to learn a preferred path to the goal first. Then this path was blocked and the pigeon might get into a period of path adjustment, exploring in the maze to find a new path to the goal. Finally, they recovered from adjustment by learning a new path

In our experiments, six pigeons (P090, P094, P097, P100, P110, and P130) were implanted electrode arrays in Hp (Fig. 1a), and we trained them to perform the above goal-directed spatial cognitive task (Fig. 2a–d). We found that the behavioural performance process of all pigeons could be divided into three phases, Phase 1 (acquisition), Phase 2 (adjustment), and Phase 3 (recovery). We defined each experimental period including the above three phases as a session and finally obtained a total of 16 sessions for all pigeons (*n* = 6; s = 6 sessions for P090, s = 1 session for P094, s = 1 session for P097, s = 3 sessions for P100, s = 4 sessions for P110, s = 1 session for P130), which included the different number of trials during the above three phases respectively.

For all of the sessions from six pigeons, the average time the pigeons spent and the path length the pigeons walked from the starting position to the goal under different experiment phases (acquisition, adjustment, and recovery) were calculated. We hope the statistical results can provide behavioural evidence for the division of the phases.

**Fig. 3:**
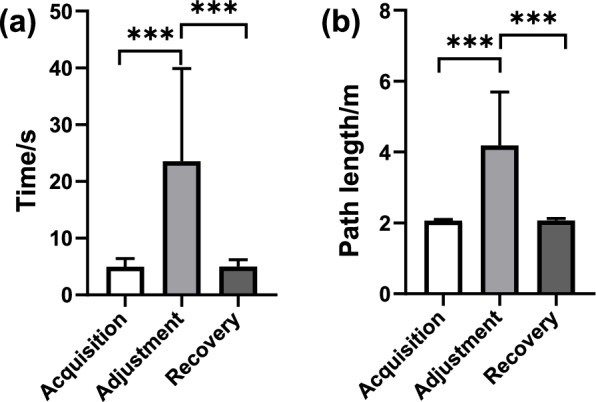
Average time spent and path length walked across all pigeons (*n* = 6 pigeons, s = 16 sessions). **A** Average time. **B** Average path length. Error bars indicate standard error. Significant differences are indicated by star marks (* *p* < 0.05, ** *p* < 0.01, *** *p* < 0.001, rank-sum test)

The time spent from the starting position to the goal could reflect the familiarity of the animal with the current environment, and it can be used as a behavioural indicator to measure their spatial cognitive function. We recorded these durations during the acquisition, adjustment, and recovery phases in each session. For each phase, we calculated the mean values and standard errors and statistically analyzed the results (Fig. 3a). The results reveal that the total duration from the starting position to the goal can indicate the different three phases, which are significantly longer in the adjustment phase than acquisition and recovery phases across all pigeons (*p* < 0.001, Fig. 3a), while the duration of the phases during acquisition and recovery phases show no significant difference (*p* > 0.05, Fig. 3a).

The path length from the starting position to the goal pigeons walked can be used as another behavioural indicator to measure their spatial cognitive function. Similarly, we recorded these path lengths during three different phases across all pigeons. For each phase, the mean values and standard errors were calculated and the results were analyzed statistically (Fig. 3b). Similar to the results of the durations, the results of path lengths from the starting position to the goal also can be used to represent the different three phases. For all of the pigeons, the path lengths of the adjustment phase are significantly longer than the other two phases (*p* < 0.001, Fig. 3b), while the path lengths of the phases during acquisition and recovery show no significant difference (*p* > 0.05, Fig. 3b).

### Hippocampal functional connectivity during the different phases

Synchronization of neural oscillations in a specific brain region has been proposed to be used for the representation of its function. Pigeons may be in different cognitive states in each of the above phases, which could be measured by the functional connectivity pattern in the brain. Therefore, we asked whether LFP functional connectivity in Hp was modulated by the goal-directed spatial task acquisition. We recorded LFPs in the Hp of the pigeons when they performed the spatial cognitive task. Specifically, we constructed the functional networks for the different frequency bands LFPs based on the coherence coefficient matrix to analyze the functional connectivity of the pigeon’s Hp in different rhythms during the acquisition, adjustment, and recovery phases in each session. We hope that these neural results will help us to understand the brain network connectivity pattern under different cognitive states further and provide more neural evidence from inner the brain for spatial path learning and adjustment.

**Fig. 4:**
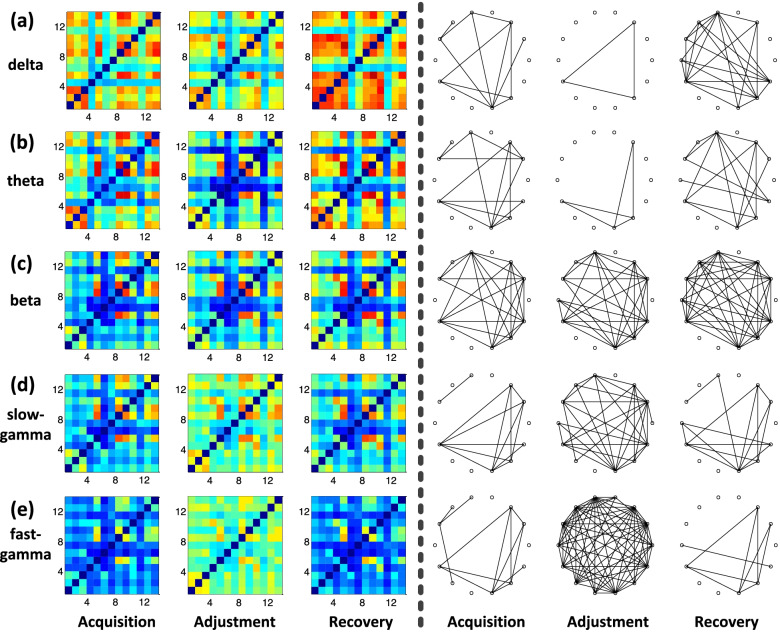
The heatmaps of the coherence coefficient matrices and binarized functional networks for different sessions in the different bands (results of P100 as the examples). **a** Delta band. **b** Theta band. **c** Beta band. **d** Slow-gamma band. **e** Fast-gamma band. (Left) Heatmaps, in which the rows and columns indicate the indexes of the channels. The color represents the coherence coefficient between any two channels. As the coefficient becomes higher, it appears to turn red and conversely turn blue. (Right) Binarized functional networks, in which each hollow circle indicates a channel

To explore the functional connectivity of the hippocampal networks during the different phases (acquisition, adjustment, and recovery), we calculated the coherence coefficients of the LFPs between all channels and visualized the corresponding matrices heatmaps in different bands across all sessions first (results of P100 as the examples are shown in Fig. 4), in which the bluer the color in the functional connection matrix, the smaller the connection weight in Hp. And the more the blue pixels in the functional connection matrices, the sparser the connections of the functional networks. Then, we binarized these above matrices to visualize the network connections more concisely and clear.

It can be seen intuitively that during the phase of adjustment, the functional connection strengths of Hp in delta and theta band are seemingly lower than those during the phases of acquisition and recovery (Fig. 4a and b, Left). Meanwhile, the binarized functional network connections of Hp during adjustment in these two bands also show a more obvious trend of sparsity than the other two phases (Fig. 4a and b, Right). However, the heatmaps of the coherence coefficient matrices and binarized functional networks results in the two gamma bands indicate that the hippocampal connection strengths in gamma bands are highest during adjustment (Fig. 4d and e, Left). And the binarized networks can also guide us to the opposite conclusion that the hippocampal connectivity shows a densification trend compared with the phases of acquisition and recovery (Fig. 4d and e, Right). Besides, for the beta band, we integrate the related results but identify no significant changes in either the heatmaps of the coherence coefficient matrices and binarized functional networks between different phases (Fig. 4c).

**Fig. 5:**
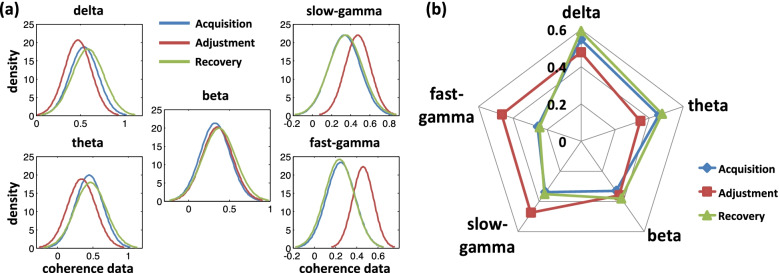
Fitted normal density distribution curves and radar map of their corresponding expected values estimated from the coherence matrices data during three phases of different bands (results of P100 as the examples). **a** Fitted distribution curves. **b** Radar map of expected values

The visualization of the coherence matrices and the binarized networks show that the connections in Hp during the path adjustment phase tend to be sparser in the lower bands while more densified in the higher bands compared with the other two phases from the qualitative point of view. This is also quantitatively evident when the fitted normal density distribution curves estimated from the coherence matrices data are plotted across the different phases (Fig. 5a). Specifically, the distributions of the curves in delta and theta bands during adjustment bias toward smaller data values compared with the other two phases, while for the two gamma bands, the distributions of the curves during adjustment bias toward higher data values. And for all bands, there is no obvious bias between the curve distributions of the phases during acquisition and recovery. Next, we tested whether the expected values of the curve distributions during different phases were specific to some particular band. For this, we plotted all of the expectations into a radar map. Indeed, lower expectations in delta and theta bands and higher expectations in two gamma bands were selectively detected during the adjustment phase (Fig. 5b).

For further quantitative analysis on all pigeons, we next tested whether the changing functional connectivity was significantly specific to some particular phase of the task. The topological properties, clustering coefficient (Coef) and global efficiency (Eff), of the networks in different bands across all sessions were calculated and analyzed statistically from the perspective of graph theory.

**Fig. 6:**
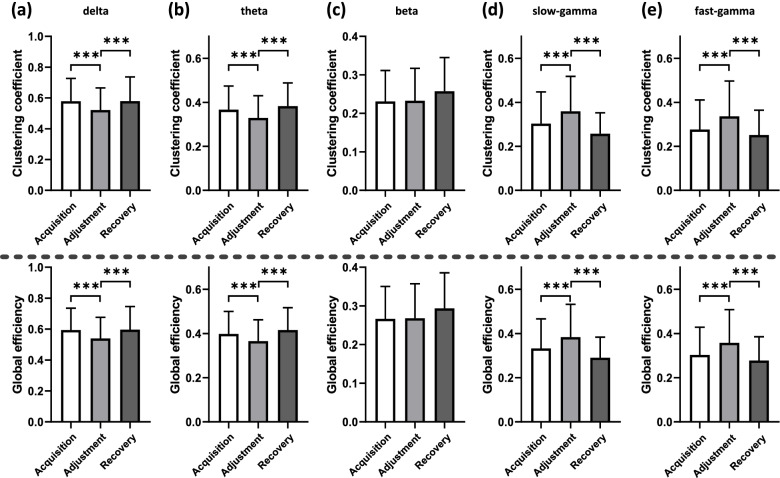
Comparative analysis of network topological properties under different states for all sessions in the different bands (*n* = 6 pigeons, *s* = 16 sessions). **a** Delta band. **b** Theta band. **c** Beta band. **d** Slow-gamma band. **e** Fast-gamma band. (Top) Clustering coefficient. (Bottom) Global efficiency. Significant differences are indicated by star marks (* *p* < 0.05, ** *p* < 0.01, *** *p* < 0.001, rank-sum test)

We identified depressed functional connectivity in lower bands (delta and theta) and elevated connectivity in higher bands (slow-gamma and fast-gamma) during the phase of spatial path adjustment compared with the other two phases. Specificity, for the delta band, it can be seen that mean Coef and Eff values during the phase of adjustment are significantly lower than those during the other two phases (*p* < 0.001, Fig. 6a). And importantly, we can identify similar changes in the theta band (*p* < 0.001, Fig. 6b). The disarrangement and reorganization of the hippocampal functional connectivity during path adjustment indicate the potential value of theta band for spatial learning representation to some extent, suggesting that theta oscillations play important role in spatial cognition function.

Moreover, we did not identify functional connectivity changes between different spatial cognitive states in the beta frequency band (*p* > 0.05, Fig. 6c), showing the lack of dependence between cognitive state representation during spatial learning and adjustment and hippocampal beta connectivity. However, we identified significant changes of the two topological network properties between different phases in the gamma band (Fig. 6d and e), which show the complete opposite changing trend in low-frequency bands. Specificity, we found both mean values of the Coef and Eff during the adjustment phase were significantly higher than those during acquisition and recovery phases in slow-gamma (*p* < 0.001, Fig. 6d) and fast-gamma band (*p* < 0.001, Fig. 6e), which is consistent with the related studies suggesting that gamma oscillations are probably important for network processes of spatial cognition function. We can include that the hippocampal functional disarrangement and reorganization of the connectivity also exist in the gamma band during path adjustment while sharing dissimilar changing trends with the lower bands.

All of the above analysis shows that spatial learning and adjustment processes correlate with hippocampal functional connectivity in the specific band, suggesting that the connectivity in this region as defined by spectral coherence is likely to result from behavioural changes during spatial learning. The disarrangement of the hippocampal functional connectivity of pigeons is induced during the spatial path adjustment, in which the depressed connectivity in lower bands and elevated connectivity in gamma bands are observed. While in the recovery phase, the Hp reorganizes its local functional connectivity after learning a new path, in which the connectivity performances in these bands return to their original levels. Overall, these data suggest that spatial learning and adjustment are associated with the selectively changing of hippocampal connectivity in the specific frequency band during the goal-directed spatial navigation.

## Discussion

Our results have shown that the functional connectivity in Hp tends to be sparse in lower bands (delta and theta) during the path adjustment phase. Theta band broadly distributed across the brain has been inextricably linked to high-level cognitive processes, such as memory encoding and retrieval, working memory retention [34]. In subsequent decades after the theta oscillation was first discovered in Hp, a series of researches have shown that it supports spatial-related information encoding. Theta rhythms provide a mechanism to coordinate related activities converging in Hp from multiple areas of the brain, especially when spatial information is taken in from the environment [35]. Previous discovery of theta in birds also suggests that the synchronization of hippocampal activity in the theta range may be an ancestral feature of hippocampal organization and function during exploratory behaviour [36]. Therefore, we have reason to believe that accompanied by the change of the navigational spatial environment, the theta band activities will change. And the results of our study provide evidence for this conclusion. Thanks to the important role of theta oscillations supporting the formation, encoding, and retrieval of the spatial memory, the functional connectivity of theta band maintains at a high level when the pigeons skillfully learned the path to the goal in our study, whether during the phases of acquisition and recovery. However, during the phase of adjustment, the change of environment makes the previously established inner spatial representation in the pigeon’s brain invalid, which leads to the loss of the theta connectivity. Our results provide supports for previous researches and suggest the important role and potential value of the theta band for spatial cognition to some extent, especially where path adjustment is concerned. However, there is still another possible potential explanation for the depressed delta and theta connectivity during path adjustment. The neural changes during adjustment might also related to the fact that encountering an obstruction in the path might cause the birds to become more alert. Also, this kind of alertness might be related to the pigeon’s attention during different phases of the behavioral task.

Contrary to the results in low bands, hippocampal connectivity is enhanced in gamma bands during path adjustment. The accompanying gamma connectivity strengthening observed is consistent with the previously suggested role of gamma oscillations in spatial cognition. Gamma frequency oscillations have been suggested to underlie various cognitive functions. As the other type of intensively discussed oscillation in the studies of spatial cognition in Hp, gamma is thought to interact with theta to temporally organize theta sequences [37]. A prominent feature of the hippocampal theta rhythm is its cooccurrence with bouts of the gamma frequency band [38, 39]. In our study, when the pigeon faces the requirement of path adjustment to solve the spatial task with the detour paradigm, it needs to mobilize more attention to integrate extra novel spatial-related information for the execution of the task in the new environment. During this process, hippocampal gamma connectivity is elevated in our study. These results are consistent with the previous studies showing that gamma frequency plays important role in attention [40] and information integration [41].

Different from the above results, we have not observed any significant connectivity changes in the beta band during different phases of the experiment, even though previous studies have indicated that beta band activity seems related to the maintenance of the current sensorimotor or cognitive state [42, 43]. Furthermore, several researches have also been suggested on how oscillations may interact across different frequencies. And the coordination of neuronal oscillations generated at different frequencies has been hypothesized to be an important feature of integrative brain functions [42]. For example, it has been proposed that nesting of theta and gamma oscillations may provide a mechanism for sequential encoding of processed items in memory recall [39], temporal coding [44], and spatial representation [37]. The nesting of fast and slow oscillations is also believed to facilitate cross-modal interaction between multisensory areas [45]. In this sense, the role of the coordinated activities in different bands for the representation of path learning and adjustment is still unclear and remains to be explored further.

Furthermore, the field potentials in other bands, for example, the sharp-wave ripples (SWRs), were not reported in hippocampal recordings of pigeons while awake and immobile [36, 46]. Moreover, anesthetized bird study indicated that the Hp did not show slow-waves, nor SWRs, but instead displayed localized gamma activity [47]. However, a recent study found mammalian-like SWRs activity in the Hp of titmouse, a food-caching bird [48], regardless of whether these events originate in the Hp itself. These findings suggest that hippocampal circuit mechanisms are similar, but not quite identical between birds and mammals. Hence, future studies will be needed to determine whether different kinds of birds share same or similar SWRs representation patterns during various behaviours, which will also be one our next further research directions.

On the other hand, although our electrophysiological results suggest that hippocampal activities might play a critical role in modulating spatial learning when the environment changes suddenly, like any neurological process, one brain region is not solely responsible for complex spatial processing independently. A large number of researches have shown that the prefrontal cortex (PFC) inextricably links to complex high-level cognitive functions and allows for flexible, adaptive, and goal-directed behaviour. PFC constitutes a core element as a bridge in the distributed cortical network that links the environmental perception and action execution [4]. In the spatial navigation tasks, the agent needs to respond to the changing environment in real-time to support flexible decision-making, necessarily requiring the participation of PFC. As a comprehensive decision-making related brain region similar to mammalian PFC, the nidopallium caudolaterale (NCL) of the pigeon has a large number of projections and pathway connections with many other regions, which confirmed its key role as the convergent region. NCL is considered to be the information integration center, which functionally links associative sensory with motor areas [49]. Furthermore, it is believed that Hp and NCL may process different types of information related to different phases of navigational homing behaviour. Previous study have concluded that the avian Hp does not appear to be ideally suited for processing and temporarily storing highly associational information. While NCL, as the recipient of multimodal secondary sensory information, seemingly the prime candidate [46].

Our previous study has showed that route formation during spatial learning in goal-directed behavior is associated with enhanced Hp-NCL functional connectivity, as well as with increased hippocampal connectivity and decreased connectivity in NCL [50], suggesting the important role of NCL in route planning. An EEG study investigated the potential relationship between the neural changes and particular visual landmarks when homing from a familiar release site. Different frequency activities were found to be associated with visual perception and information processing at a higher level [51]. Although the neural signals recorded in this study are not of high spatial resolution (from a wide range of brain regions including visual wulst and NCL), it also suggests that a wider range of brain regions are involved in spatial cognition correlation during navigation Information processing. Based on these research outcomes, we predict that the activities in the NCL (even more other relevant brain regions) of the pigeon during spatial learning may correlate with the behavioural differences and the cognitive states in our tasks. This will be another meaningful issue that is worth exploring further.

## Conclusions

In this study, we explored the behavioural response of the pigeons and the functional connectivity properties of LFPs recorded from Hp during a goal-directed spatial cognitive task with a detour paradigm. We found the pigeons progressively learned to solve the path adjustment task as we predicted in the immediate aftermath of the spatial environment change, in which the preferred path the pigeons were trained to learn was blocked. Compared with the phases of acquisition and recovery, both the total duration and the path lengths pigeons walked from the starting position to the goal were significantly longer in the phase of adjustment. Moreover, the functional network analysis results of Hp shown varying connectivity changes in different bands between different phases, indicating that the path learning and adjustment processes were associated with changed hippocampal functional connectivity, especially the depressed connectivity in lower bands and elevated connectivity in gamma bands. We can conclude that we have shown that behavioural changes during path adjustment are accompanied by modifications in functional connectivity in the hippocampal network, featuring both the behavioural response and neural representation of avian spatial cognitive learning process from two levels including behaviour and neuroelectrophysiology. Altogether, our results provide insight into the selective changes of the hippocampal connectivity during path adjustment in a goal-directed spatial task of pigeons. The functional disarrangement and reorganization of the connectivity in avian Hp during different phases may contribute to our further understanding of the potential mechanism of path learning and adjustment, and also provide us the comparative and reference values to compare with other species.

## Methods

### Subjects, surgery, and electrode implantation

Pigeons weighing 400–500 g were obtained from a local supplier (Gongchuang Pigeon Co., Henan, China) and housed in an animal facility with the size of 3 m × 3 m × 2 m for at least 2 weeks before the experiments, which is with plenty of sunlight, good ventilation, and free access to water and food. After completion of all the behavioral experiments and recordings, each animal was humanely euthanized for further histologically examination of the recording locations. Each animal was given a lethal overdose of anesthetic (1.5% pelltobarbitalum natricum) and the tips of recording electrodes in Hp were marked by electrolytic lesions (5 mA for 20 s). Then, each was perfused transcardially with saline followed by 4% paraformaldehyde (prepared in 0.1 M phosphate buffered saline, pH 7.4) to get the brain according to the animal welfare regulations. Finally, frozen coronal sections (50 μm) were collected from the recording sites to be histologically examined. Every effort was made to minimize animal pain, suffering and distress and to reduce the number of animals used. No unexpected mortality or adverse events were observed. All of the experiments were conducted by individuals the Animals Act, 2006 (China), for the care and use of laboratory animals, and approved by the Life Science Ethical Review Committee of Zhengzhou University.

All surgeries were performed after the pigeons were anesthetized with 1.5% pelltobarbitalum natricum (0.25 ml/100 g) injected intraperitoneal. The pigeon was placed in a stereotaxic apparatus and the recording microelectrode array (16 channels: 4 × 4 array, Hong Kong Plexon Inc., Hong Kong, China) was chronically implanted at a location directly in the left Hp (AP 4.5 mm; ML 1.0 mm; DV 0.5–1.5 mm) according to coordinates obtained from the Karten and Hodos stereotaxic atlas of the pigeon brain [52]. The implanting location of Hp, the microelectrode array, and a pigeon with the implanted electrode are shown in Fig. 1.

### Goal-directed spatial task and apparatus

After the recovery period of about one week, the pigeons implanted with electrode arrays were taken to carry out the experiment and signal recording in the daytime every day, in which the order of experiments, measurements and caging of animal was random to minimize confounders. In our experiments, the pigeons were trained to carry out a goal-directed spatial cognitive task in a maze (Fig. 2a and b). The maze included a starting position as the waiting area, two alternative goal positions with food. At these above three positions, there were three food hampers providing food rewards. There are multiple optional paths between the starting position and the goal positions. The infrared detectors distributed on all of the pathlets along the paths were used to define the beginning and end time for signal segmentation. The gate was set at the exit of the starting position to control the beginning and end of a trial. All of the designs are used for the simulations of the processes including spatial learning and path adjustment.

In the goal-directed spatial cognitive task, the pigeons were trained to walk from the starting position to the goal location and get food rewards. It is noted that for each pigeon, one of the two alternative goals is chosen to designate as the goal location randomly, in which the randomisation lists are computer generated. Only the investigator is aware of the participant’s goal allocation. At the beginning of the trial, the hamper was opened to provide food in the goal location, and the pigeons were trained to learn a preferred path to the goal. If the pigeon arrived at the goal, this trial was recorded as a correct one. After enjoying the food reward, they were trained to go back to the starting position to start the next trial. The experiment procedures in one session could be divided into three phases (Fig. 1 c and d), while each phase was composed of multiple trials. When a pigeon could reliably perform the trial through a preferred path, in which more than 90% of the total trial numbers are corresponding to the same path on two consecutive days, it was considered that the experiments of Phase 1 (acquisition) were completed. Then the experiments entered Phase 2 (adjustment), in which the learned path was blocked at the pathlet close to the goal. In this phase, the pigeon needs to finish the path adjustment by exploring the maze to find a new path to the goal. Normally, the pigeons could learn a new path after multiple trials of training. Finally, at the end of the experiment is Phase 3 (recovery). The pigeons adapted to the new environment after the barricading, and they learned a new path to get the rewards at the goal position.

### Behavioural data and LFPs recording

The behavioural data of the pigeons was recorded by the observation camera placed on the ceiling and stored in the computer during the experiment. The behavioural trajectories and timings of the pigeons were analyzed to obtain the time the pigeons spent and the path length the pigeons walked from the starting position to the goal.

A 128 channel Cerebus™ Multichannel Acquisition Processor (Blackrock Microsystems, Salt Lake City, UT, USA) was used to record local field potential (LFP) signals from the Hp region of the pigeon. The LFPs with a sampling rate of 2 kHz were filtered by a 0–250 Hz Butterworth low-pass filter.

### Signals of interest segmentation

In our experiments, we recorded the LFPs in the Hp of the pigeons when they performed the goal-directed spatial cognitive task. To eliminate the negative influence of bad channels caused by detached electrode contacts, intermittent electrical connection, or line noise, we selected the channels with high reliability from all 16 ones of different pigeons. Then the signals of interest (SOIs) corresponding to different states from the three phases of the experiments were segmented. Finally, for each SOI segment, we filtered the LFPs and obtained the signals corresponding to the following six bands, including delta (1–4 Hz), theta (5–12 Hz), beta (13–30 Hz), slow-gamma (31–45 Hz), and fast-gamma (55–80 Hz).

### Functional network analysis

The brain can be considered as an extremely complex network and functional connectivity is a powerful tool used to characterize the brain networks in the local brain region. To obtain the mathematical representation of the brain, the nodes and edges are defined based on complex networks theory in brain functional network analysis. Then, the correlation intensity between the network nodes can be measured by the incident matrix. As a commonly used criterion to construct an incident matrix, coherence [53] can be used not only to analyze the degree of the synchronization but also to represent the linear or interdependent relationship of the variables in the frequency domain, which is defined as the normalized result of two independent signals. In this paper, we used coherence to measure the relationship between the LFPs corresponding to the channels in Hp, and the coherence coefficient is calculated as follows:

1 $${Coh}_{x,y}(f)=\frac{{\left|{p}_{x,y}(f)\right|}^2}{\left|{p}_x(f)\right|\times \left|{p}_y(f)\right|}$$

where

2 $${p}_{x,y}(f)=\frac{1}{n}\sum \limits_{i=1}^n{x}_i(f){y}_i^{\ast }(f)$$

For a given frequency *f*, *p*_*x*_(*f*) and *p*_*y*_(*f*) represent the auto-power spectrums of two LFP time series *x* and *y* respectively, and *p*_*x*, *y*_(*f*) is the cross-power spectrum. In this paper, we calculated the LFPs coherence of each pair of channels from all channels in Hp. The coherence matrices of different frequency bands were obtained to construct the functional network. In our work, the channels in the Hp can be defined as the nodes of the network and the connection between any two nodes can be defined as the edge of the network. We can binarize the above coherence matrices based on the appropriate thresholds to realize the visualization of the network connections [54].

The topological characteristics of the brain functional network can be used to reflect the functional connectivity of the brain and help to reveal the cognitive state in the brain. In this paper, we selected two representative characteristics, clustering coefficient (Coef) and global efficiency (Eff) for statistical analysis of the networks [55]. The clustering coefficient reflects the intensity of the connection between different nodes (channels in Hp) in the functional network. The calculation formula is as follows:

3 $$\mathrm{Coef}=\frac{1}{M}\sum \limits_{i=1}^M\frac{2{E}_i}{k_i\left({k}_i-1\right)}$$

where *k*_*i*_ indicates that the *i*-th node has *k* edges connected with the other nodes. *E*_*i*_ indicates the number of edges in the network that connects with the *i*-th node. *M* is the total number of nodes in the network. The value of Coef ranges from 0 to 1. The larger the value is, the closer the information between the two nodes in the network will be.

Global efficiency can be used not only to measure the efficiency and ability of the information transmission and processing in the brain functional network but also to reflect the degree of network integration. It is defined as:

4 $$\mathrm{Eff}=\frac{1}{M}\sum \limits_{i\in M}\frac{\sum_{j\in M}{\left({d}_{ij}\right)}^{-1}}{M-1}$$

where *d*_*ij*_ indicates the shortest path length between two nodes *i* and *j*.

### Statistical analysis

All statistical analyses were performed by Matlab R2014a software (The MathWorks, Inc., Natick, MA, USA), using the rank-sum test. Statistical results were presented as mean ± standard deviation (std), and the statistically significant difference level was set to 5%. Statistically significant were indicated by *p* value as follows: * *p* < 0.05, ** *p* < 0.01, *** *p* < 0.001.

## Data Availability

The datasets used during the current study will be available from the corresponding author on reasonable request.

## Abbreviations

Hp: Hippocampus
SWRs: Sharp-wave ripples
AP: Anteroposterior
ML: Mediolateral
DV: Dorsoventral
LFP: Local field potential
SOI: Signals of interest
Coef: Clustering coefficient
Eff: Global efficiency
std.: Standard deviation
PFC: Prefrontal cortex
NCL: Nidopallium caudolaterale
